# Single Nucleotide Polymorphisms and Post-operative Complications Following Major Gastrointestinal Surgery: a Systematic Review and Meta-analysis

**DOI:** 10.1007/s11605-019-04300-2

**Published:** 2019-07-03

**Authors:** Joseph Beecham, Andrew Hart, Leo Alexandre, James Hernon, Bhaskar Kumar, Stephen Lam

**Affiliations:** 1 Norwich Medical School, University of East Anglia, Norwich, NR4 7TJ UK; 2 Norfolk and Norwich University Hospitals NHS Foundation Trust, Norwich, NR4 7UY UK

**Keywords:** Post-operative complications, Genetics, Gastrointestinal surgery

## Abstract

**Background:**

The human genome is an under-researched area of pre-operative risk stratification. Studies of genetic polymorphisms and their associations with acute post-operative complications in gastrointestinal surgery have reported statistically significant results, but have varied in methodology, genetic variations studied, and conclusions reached. To provide clarity, we conducted a systematic review and meta-analysis of single nucleotide polymorphisms and their association with post-operative complications after major gastrointestinal surgery.

**Methods:**

We performed a literature search using Ovid MEDLINE and Web of Science databases. Studies were included if they investigated genetic polymorphisms and their associations with post-operative complications after major gastrointestinal surgery. We extracted clinical and genetic data from each paper and assessed for quality against the STrengthening the REporting of Genetic Association Studies (STREGA) guidelines. Odds ratios were presented, with 95% confidence intervals, to assess strengths of association. We conducted a meta-analysis on *TNF-α-308*, which had been assessed in three papers.

**Results:**

Our search returned 68 papers, of which 5 were included after screening and full-text review. Twenty-two different single nucleotide polymorphisms (SNPs) were investigated in these studies. We found that all papers were genetic association studies, and had selected SNPs related to inflammation. The outcome investigated was most commonly post-operative infection, but also anastomotic leak and other non-infectious complications. Statistically significant associations were found for *TNF-α-308*, *IL-10-819*, *PTGS2-765* and *IFN-γ-874.* There was significant variability in study quality and methodology. We conducted a meta-analysis on associations between the *TNF-α-308* polymorphism and post-operative infection and report an OR of 1.18 (CI 0.27–5.21).

**Conclusions:**

We found biologically plausible associations between SNPs involved in inflammation and post-operative infection, but the available data were too limited and of insufficient quality to reach definitive conclusions. Further work is needed, including genome-wide association studies (GWAS).

## Introduction

Gastrointestinal surgical pathology represents a growing challenge to healthcare systems around the world,^1,2^ with both developing and industrialised societies shouldering this burden.^1,3–6^ In the UK alone, 1.3 m general surgical interventions take place annually.^7^ Unfortunately, major gastrointestinal surgery is accompanied by complication rates of up to 24%.^8^ Some 220,000 hospital admissions were due to complications from surgery in 2017–2018 in the UK alone.^9^ The commonest post-operative complication is infection, but others include cardiac arrhythmias and acute kidney injury, as well as anastomotic leakage and wound dehiscence.^10^ Although these have been ameliorated by advances in critical care, minimally invasive techniques, subspecialisation and centralisation of expertise, improved risk stratification may inform future interventions to reduce risk. Quantifying these risks accurately is ever more important as we face decisions to operate on a population that is increasingly older with significant co-morbidities.

Current perioperative risk management involves well-established tools such as P-POSSUM and ASA grades.^11^ However, these tools involve subjective judgement, and there is evidence that P-POSSUM scoring in particular overestimates risk.^12–14^ The genome’s contribution to post-operative complications is under-researched and may better explain variance which is unaccounted for by clinical assessment tools. For instance, there is convincing research linking genetic variation with the host response to sepsis and trauma, but there is limited work investigating this in surgery.^15–18^ Single nucleotide polymorphisms (SNPs) are the commonest form of genetic variation,^19^ and many have been linked to disease.^20^ For instance, polymorphisms associated with high *TNF-α* have been associated with sepsis.^18,21^*TNF-α* is a critical cytokine regulating acute inflammation via activation of immune cells, release of large amounts of inflammatory mediators, downstream signalling and endocrine effects.^22^

The association between particular SNPs and risk of developing post-operative complications has been explored in cardiac surgery,^23^ with one group reporting three SNPs associated with post-operative myocardial infarction.^24^ There have been several genetic association studies taking a similar approach in gastrointestinal surgery, which have aimed to identify whether particular polymorphisms are associated with poor outcome. These studies are challenging to interpret and were limited by small sample sizes, incomplete reporting of results, population stratification and implausible effects of individual SNPs, amongst others. To our knowledge, this is the first review examining these publications in a systematic manner. Such work may identify high-risk genes, which if externally validated as part of a clinical tool, could allow clinicians to offer improved risk prediction as part of the increasingly affordable ambition of personalised medicine. Addressing these risks perioperatively, with prehabilitation for example, could reduce future surgical morbidity and mortality.

## Methods

### Objectives

To systematically review studies in patients who have undergone major gastrointestinal surgery, and whether any single nucleotide polymorphisms are associated with post-operative complications.

### Protocol and Registration

We followed the guidelines of the PRISMA statement^25^ in preparing this review and registered with the international prospective register of systematic reviews, PROSPERO (CRD42019122342) on 15 February 2019.^26^

### Search Strategy

We searched the electronic databases MEDLINE (using the Ovid platform) and Web of Science for papers published from January 1990 to January 2019. The following free-text terms were used: “surgical procedures”, “gastrointestinal surgery”, “genetic polymorphism”, “single nucleotide polymorphism”, “post-operative complications” and “post-operative outcomes”. For MEDLINE searches, we used the MeSH terms “digestive system surgical procedures” AND “polymorphism, genetic” AND “postoperative complications”. Searches involving MeSH terms included index subheadings. We also reviewed reference lists of both identified articles and review articles for additional relevant studies. We did not have any language restrictions.

### Study Selection, Inclusion and Exclusion Criteria

Studies were independently screened by title and abstract by JB and SL. Both authors subsequently performed full-text review. Any disagreement was resolved by discussion with a third author. We considered a study for inclusion if it met the following criteria: (1) patients underwent major oesophagogastric, hepatobiliary and colorectal resections (elective or emergency), (2) the variation studied was a single nucleotide polymorphism, and (3) post-operative complications were investigated in the 90-day post-operative period. We excluded studies in less major operations, including cholecystectomy, appendectomy and hernia repair. We also excluded outcomes which were not strictly complications, such as analgesic requirement and risk of cancer recurrence. Participants with particular conditions including inflammatory bowel disease, transplant patients and immunocompromised individuals were also excluded.

### Data Extraction and Synthesis

For each eligible study after full-text review, we extracted year of publication, study population, sample size, patient demographics, post-operative outcomes, outcome measure, statistical analyses and statistic presented. Genetic information extracted included frequency of alleles, SNPs of interest, SNP reference identifier (where published), genotype method and locus.

### Risk of Bias and Quality Assessment

Each study was assessed for quality using the STrengthening the REporting of Genetic Association Studies (STREGA) addendum to the Strengthening Reporting of Observational Studies (STROBE) statement.^27^

### Statistical Analysis

Where multiple studies (defined as ≥ 3) reported on the same SNP, a meta-analysis was conducted. The analysis, as well as tests of heterogeneity, and Hardy-Weinberg calculations were performed using the MetaGenyo: Meta-Analysis of Genetic Association Studies tool.^28^ Our *p* value threshold for Hardy-Weinberg equilibrium was 0.05. If the included studies demonstrated significant heterogeneity, a random effects model was applied. Heterogeneity was assessed with a *I*^2^ statistic with values of 25% considered low, 50% moderate and 75% high. Egger’s test assessed for publication bias.

## Results

### Study Identification, Exclusion and Inclusion

**Fig. 1:**
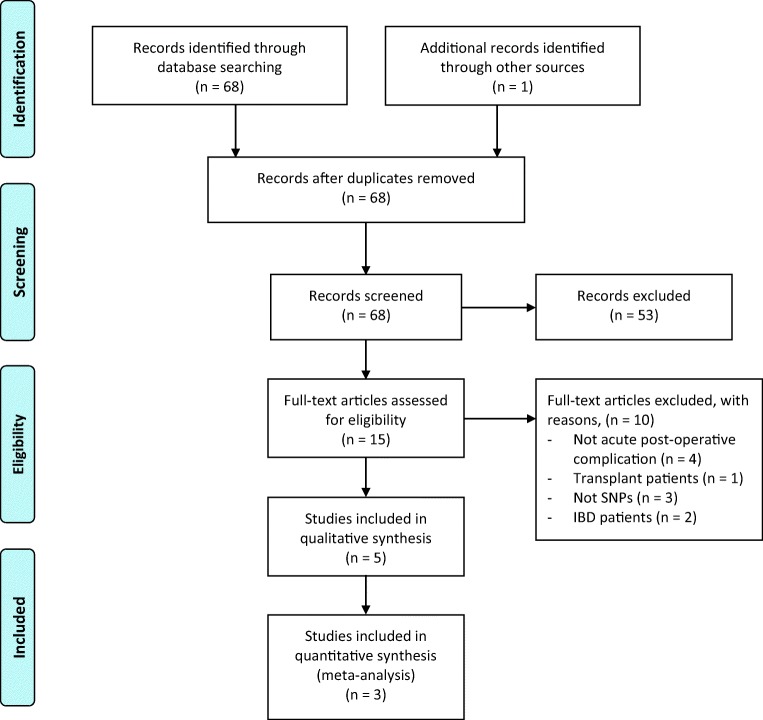
Flow chart of study identification, exclusion and inclusion. Adapted from the PRISMA statement^25^

Sixty-eight studies were identified through electronic searches of databases (Fig. 1), and one additional study was identified through hand-searching. There was a single duplicate. Sixty-eight records were screened by title and abstract, and 53 were excluded at this stage. Most exclusions were due to study participants being transplant patients. Fifteen full-text papers were assessed for eligibility and two were excluded due to participants with inflammatory bowel disease^29,30^ and one paper excluded for participants who had undergone transplant surgery.^31^ A further three were excluded due to reporting non-SNP sources of genetic variation, such as insertion/deletion (indel) polymorphisms,^32–34^ and four for reporting outcomes which were not of interest.^35–38^ This resulted in five remaining studies.^39–43^

### Study Designs, Sample Sizes and Data Analysis

Table 1 shows the main characteristics of the included studies. Two papers were from a Japanese population,^41,42^ the remainder were Irish,^39^ Indian^40^ and Dutch.^43^ All broadly used a case-control design and all secured ethical review. The vast majority of SNPs examined were from genes involved in inflammation. Two papers also measured pro-inflammatory cytokine levels.^40,42^ One of the papers looked predominantly at mouse anastomotic leakage but reported the results of a small SNP analysis in humans.^43^ Samples sizes ranged from 110 to 239 patients. The number of SNPs examined per study ranged from 1 to 15. Every study reported statistically significant findings. Three of the studies investigated solely infection as the primary outcome,^40–42^ whilst one a broad range of complications^39^ and one examined anastomotic leak.^43^ Three studies were in patients undergoing oesophagectomies,^39,41,42^ one in colectomies^43^ and one study examined a broad range of gastrointestinal surgical procedures.^40^

**Table 1:** Characteristics of included studies

First author	Year	Operation	Population	Patient sample size	SNPs	Genotype method	Statistic	Outcomes	Infection criteria
Azim, K^39^	2007	Oesophagectomy	Irish	197	TNF-⍺-238 TNF-⍺-308 TNF-⍺-857 TNF-⍺-863 IL-1β-31 IL-1β-511 IL-1β + 3953 IL-1β 5200 TLR4 + 896 IL-10-592 IL-10-819 IL-10-1082	TaqMan	*χ*^2^ Fisher’s exact Odds ratio	Infectious and non-infectious complications	Sepsis: SIRS + blood cultures Pneumonia: either positive culture or clinical or radiological evidence of consolidation
Motoyama, S^41^	2009	Oesophagectomy	Japanese	110	IFN-γ-874 TNF-⍺-238 TNF-⍺-308 TNF-⍺-1031 TNF-β 250 TGF-β1 29 IL-1β-31 IL-1β-511 IL-2-330 IL-4-590 IL-6-634 IL-6 receptor 48892 IL-10-592 IL-12β-1188	PCR-RFLP	Pearson *χ*^2^ Fisher’s exact Odds ratio	Infectious and non-infectious complications	Sepsis: positive blood cultures Pneumonia: positive culture and radiologic evidence of consolidation
Baghel, K^40^	2014	Multiple GI surgeries	Indian	239	TNF-⍺-308	TaqMan	Pearson *χ*^2^ Fisher’s exact Odds ratio	Infection	Sepsis: SIRS
Sakamoto, K^42^	2014	Oesophagectomy	Japanese	120	TNF-⍺-1031 IL-1β-511 IL-6-634 IL-10-819	ARMS-PCR	Pearson *χ*^2^ Fisher’s exact Odds ratio	Pneumonia	Pneumonia: pyrexia > 38 °C and either positive sputum cultures or clear clinical or radiological evidence of consolidation
Reisinger, K^43^	2017	Colectomy	Dutch	148	PTGS2-765	PCR-RFLP	Pearson *χ*^2^	Anastomotic leak	Not applicable

### Technical Weaknesses

Methodological quality was highly variable. Most papers did not explain how their sample sizes were calculated. Two papers failed to report on Hardy-Weinberg equilibrium.^40,43^ Most papers did not use a standardised method of defining the SNP of interest (such as *rs* number). Although most papers used accepted definitions of surgical operations, one study used a broad definition of major gastrointestinal surgery and did not specify the precise procedures undertaken.^40^ One paper failed to report participant details such as age, gender or ethnicity.^43^ One paper did not report a full breakdown of genotype distributions.^42^ Two of the studies examined the same SNPs but came to opposite conclusions.^39,40^ There was highly variable reporting of laboratory methods of genetic analysis.

### SNP Associations—Inflammation and Infection

All five papers examined polymorphisms associated with inflammation, as illustrated in Table 2. Four reported a positive association with an infectious complication,^39–42^ and one paper found an positive association with anastomotic leak.^43^ One of the papers examined, but did not find, a link with non-infectious complications.^41^

**Table 2:** Studies demonstrating SNPs statistically significantly associated with infection

Study	SNP	Gene function	Reported genetic association	Reported statistic
Azim, 2007^39^	TNF-α-308	Pro-inflammatory cytokine	A allele associated with a reduced risk of post-operative infections	*p* = 0.017
Baghel, 2014^40^	TNF-α-308	Pro-inflammatory cytokine	A allele associated with an increased risk of post-operative sepsis	*p* = 0.037
Motoyama, 2009^41^	IFN-γ-874	Pro-inflammatory cytokine	AT genotype associated with an increased risk of post-operative infections	*p* = 0.0215
Reisinger, 2016^43^	PTGS2-765	COX-2 gene, pro-inflammatory function	CC genotype associated with an increased risk of post-operative anastomotic leak	*p* = 0.02
Sakamoto, 2014^42^	IL-10-819	Anti-inflammatory cytokine	TT genotype associated with an increased risk of post-operative pneumonia	*p* = 0.0323

Twenty-two different SNPs in total were reported in these studies. Only two SNPs were investigated by three or more studies and eight SNPs were investigated by two more studies. The studies reported statistically significant associations for the following SNPs: *TNF-α-308*, *IL-10-819*, *PTGS2-765* and *IFN-γ-874* (Table 1)*.*

Three studies reported results on *IL-1β-511,*^39,41,42^ another mediator of inflammation. One of these did not report allele distributions,^42^ so was unsuitable for a quantitative analysis.

### Meta-analysis of *TNF-α-308* Polymorphism

**Fig. 2:**
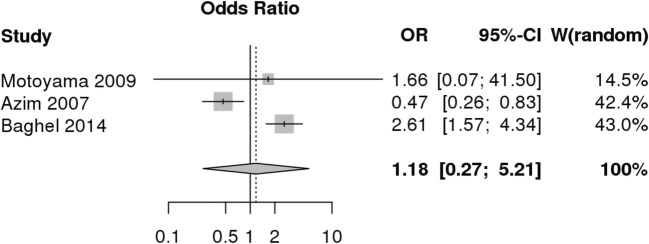
Forest plot demonstrating TNF-α-308 and risk of infection, using an allele contrast model (A vs G)

Three studies examined the effect of *TNF-α-308* (rs1800629) on infection post-operatively^39–41^ (Fig. 2). The A allele is associated with increased levels of *TNF-α* protein^44^ and is considered the risk allele. Using the MetaGenyo^28^ statistical tool, we undertook a quantitative synthesis using a random effects model. Genotype distributions and Hardy-Weinberg *p* values are shown in Table 3. We used an allele contrast model (A allele vs G allele) and a dominant model (AA + AG vs GG). A recessive model was not suitable as one of the studies had no participants with the AA genotype.^41^ The allele contrast model (A vs G) reported an odds ratio of 1.18 (CI 0.27–5.21), *p* = 0.281, as shown in Table 4. Both models reported a *I*^2^ value of over 80% and Egger’s test *p* = 0.96. In both comparison models, the meta-analysis failed to demonstrate any significant association between the polymorphism and infectious outcome. Two of the papers came to opposite conclusions about the role of the allele.^39,40^

**Table 3:** Genotype distributions and Hardy-Weinberg equilibrium in cases and controls

Study	Infectious complication			No infectious complication			
	AA	AG	GG	AA	AG	GG	HWE adjusted *p* value
Motoyama, 2009^41^	0	0	18	0	1	90	0.958
Azim, 2007^39^	0	17	38	10	60	72	0.899
Baghel, 2014^40^	8	15	24	9	43	140	0.075

**Table 4:** Association test results for TNF-α-308 and infection, random effect method

Comparison model	Odds ratio	95% CI	*p* value	*I*^2^
Allele contrast	1.176	[0.265; 5.214]	0.281	89%
Dominant model	1.155	[0.266; 5.009]	0.848	84%

## Discussion

### Key Results

This review found that research examining SNPs and post-operative complications has focussed almost exclusively on immunity and infection as clinical outcomes. We identified four polymorphisms of immune cytokine genes were associated with post-operative complications. We also conducted a meta-analysis of the only SNP which was examined by three or more studies, *TNF-α-308.* This polymorphism is involved in production of *TNF-α*, pro-inflammatory cytokine. Our meta-analysis examined the SNP in the context of gastrointestinal surgery. One study reported patients with the *TNF-α-308* GG genotype had fourfold higher odds of a post-operative infection: OR 4.2 (CI 1.7–10.5),^39^ whilst another reported that it was the AA genotype which was associated with infection with OR 4.17 (CI 1.5–11.48).^40^ We can compare this with the results of a previous meta-analysis which examined *TNF-α-308* in sepsis more generally. This found a much more conservative effect size with OR = 1.32 (*p* < 0.001) in the A vs G allele model in a much larger data set (*n* = 9373).^45^ The differing results could conceivably be explained by statistical error, for the reasons outlined below.

### Limitations

We found that the variety of SNPs identified have not been researched in an organised fashion. Despite the number of SNPs examined (22), there was little overlap between papers in terms of which SNPs were studied. There was poor consistency between findings and results were not replicated. These findings are thus highly vulnerable to type 1 and 2 error. The problem is compounded by the very small size of relevant published research. We only identified five relevant papers in total, and only three which examined non-infectious complications. As Table 1 shows, there was also variability in how infection was defined in these studies, which may have contributed to the heterogeneity of results.

Our work has demonstrated highly variable outcome measures, populations, genotyping methods and conclusions. These methodological issues are common in genetic epidemiology.^46^ Results suitable for extensive meta-analysis are essential in genetic association studies but only a minority of positive gene-association results are ever reproducible.^47,48^ Large, well-powered samples are required to examine the effect of single polymorphisms on complex multifactorial outcomes,^47,49^ particularly when the true effect size may be small. The evidence presented here shows that further meta-analysis is essential because of small sample sizes, but also challenging because too few papers examined the same SNPs.

The scattershot approach taken in the existing literature is unlikely to yield meaningful results. These papers have all been genetic association studies with SNPs pre-selected. This makes the findings vulnerable to confirmation bias.

### Interpretation and Biological Mechanisms

Despite the difficulties we have highlighted, it is biologically plausible that genetic variation leading to a dysregulated immune system could result in post-operative infection or sepsis. Sepsis is a complex entity involving aberrant host responses to an infectious pathogen.^50^ It involves a broad range of pro- and anti- inflammatory signals with mediation at the organ, tissue, cellular and molecular levels.^51,52^

*TNF-α* is a cytokine and pyrogen with a multitude of complex inflammatory effects, produced predominantly by macrophages but also by B and T lymphocytes.^53,54^ It induces downstream inflammatory pathways via nuclear factor κΒ activation and arachidonic acid formation.^55^ It has a synergistic effect with IL-1β and also has roles in apoptotic cell death, macrophage differentiation, IL-6 induction, fibroblast production, B cell proliferation and direct antiviral activity.^53^ It is also thought to have both pro- and anti-inflammatory roles depending on the phase of the acute insult.^53,55^ The *TNF-α-308* functional polymorphism has been shown to correlate with variability in TNF cytokine production,^56^ and individuals who are genetically predisposed to over-express *TNF-α* suffer more pronounced inflammatory reactions.^55^ This would be consistent with published meta-analyses linking *TNF-α* polymorphisms to sepsis generally.^18,45^

Despite the promising explanatory framework for why a cytokine polymorphism may contribute to post-operative complications, our work shows that the existing data in major gastrointestinal surgery are not clear or strong enough to draw clinically meaningful conclusions at present.

### Future Work

There are no genome-wide association studies (GWAS) published in this area. We suggest that an efficient route for answers would be for this type of study to be performed in the first instance, identifying candidate SNPs. These could then be tested in multiple large-scale genetic association studies, which could subsequently be meta-analysed.

#### Statement of Authorship

JB contributed to acquisition and analysis of data, the design and concept of the work, and drafted the manuscript. AH contributed to the conception, analysis and revisions of the work. LA, JH and BK contributed to analysis, interpretation and revisions of the work. SL contributed to the conception, acquisition, analysis and interpretation of data and revisions of the work. All authors approve of the final version of the manuscript.
